# Sustainable Microfabrication Enhancement of Graphene Nanoplatelet-Reinforced Biomedical Alumina Ceramic Matrix Nanocomposites

**DOI:** 10.3390/nano13061032

**Published:** 2023-03-13

**Authors:** Mustafa M. Nasr, Saqib Anwar, Ali M. Al-Samhan, Khaled N. Alqahtani, Abdulmajeed Dabwan, Mohammed H. Alhaag

**Affiliations:** 1 Industrial Engineering Department, College of Engineering, Taibah University, Medinah 41411, Saudi Arabia; 2 Industrial Engineering Department, College of Engineering, King Saud University, Riyadh 11421, Saudi Arabia

**Keywords:** Al_2_O_3_ matrix nanocomposites, graphene nanoplatelets, clean and sustainable manufacturing, high-frequency induction heating, laser micromachining performance, surface integrity

## Abstract

Studies about adding graphene reinforcement to improve the microfabrication performance of alumina (Al_2_O_3_) ceramic materials are still too rare and incomplete to satisfy sustainable manufacturing requirements. Therefore, this study aims to develop a detailed understanding of the effect of graphene reinforcement to enhance the laser micromachining performance of Al_2_O_3_-based nanocomposites. To achieve this, high-density Al_2_O_3_ nanocomposite specimens were fabricated with 0 wt.%, 0.5 wt.%, 1 wt.%, 1.5 wt.%, and 2.5 wt.% graphene nanoplatelets (GNPs) using a high-frequency induction heating process. The specimens were subjected to laser micromachining. Afterward, the effects of the GNP contents on the ablation depth/width, surface morphology, surface roughness, and material removal rate were studied. The results indicate that the micro-fabrication performance of the nanocomposites was significantly affected by the GNP content. All nanocomposites exhibited improvement in the ablation depth and material removal rate compared to the base Al_2_O_3_ (0 wt.% GNP). For instance, at a higher scanning speed, the ablation depth was increased by a factor of 10 times for the GNP-reinforced specimens compared to the base Al_2_O_3_ nanocomposites. In addition, the MRRs were increased by 2134%, 2391%, 2915%, and 2427% for the 0.5 wt.%, 1 wt.%, 1.5 wt.%, and 2.5 wt.% GNP/Al_2_O_3_ nanocomposites, respectively, compared to the base Al_2_O_3_ specimens. Likewise, the surface roughness and surface morphology were considerably improved for all GNP/Al_2_O_3_ nanocomposite specimens compared to the base Al_2_O_3_. This is because the GNP reinforcement reduced the ablation threshold and increased the material removal efficiency by increasing the optical absorbance and thermal conductivity and reducing the grain size of the Al_2_O_3_ nanocomposites. Among the GNP/Al_2_O_3_ nanocomposites, the 0.5 wt.% and 1 wt.% GNP specimens showed superior performance with minimum defects in most laser micromachining conditions. Overall, the results show that the GNP-reinforced Al_2_O_3_ nanocomposites can be machined with high quality and a high production rate using a basic fiber laser system (20 Watts) with very low power consumption. This study shows huge potential for adding graphene to alumina ceramic-based materials to improve their machinability.

## 1. Introduction

Ceramics have attractive properties such as high elastic stiffness, mechanical strength, biocompatibility, and stability at high temperatures, making them useful for biomedical, electronic, automotive, and aerospace applications [1]. Alumina is an example of a ceramic material that is very commonly used in a wide range of applications, such as micro-reactors [2], microfluidic devices [3], heat exchangers, heatsinks [4], and electronic substrates [3]. However, these materials have been limited by their intrinsic brittleness, high hardness, poor electrical and thermal conductivity, and poor machinability. To overcome these challenges, alumina ceramic matrix composites reinforced with nanostructure reinforcement have been developed to create new advanced materials with unique properties that cannot be obtained using a single monolithic ceramic. Earlier attempts to fabricate ceramic matrix composites have reinforced them with ceramic or metallic particles, fibers, or whiskers [5,6,7]. However, incorporating alumina ceramics with these reinforcement materials cannot satisfy the requirements of their direct use as a structural component, such as in armor [8,9,10] or dental implants [11]. With the emergence of graphene, it has become the ideal nanostructure filler for improving the toughness, brittleness, and electrical and thermal conductivity of metal/ceramic-based nanocomposites [12,13,14,15]. This is because graphene inhibits grain growth, and the network distribution of graphene reinforcement enhances ceramic conductivity [11,16].

In the recently published literature on using graphene for reinforcing ceramic-based nanocomposites, Al_2_O_3_ matrix nanocomposites have been of particular interest since they have exhibited improvements in mechanical properties and thermal and electrical conductivity compared to pure ceramic matrices [17,18,19,20,21,22]. For instance, He et al. [23] presented the first study on GNP-reinforced alumina ceramic matrix composites. Graphene was successfully incorporated into the alumina matrix using the ball-milling technique and then sintered by spark plasma sintering (SPS). They showed that adding graphene to the alumina matrix prevented grain growth during sintering, resulting in a fine-grained structure. Wang et al. [24] fabricated alumina composites with 2 wt.% GNS using mechanical stirring and SPS processes. The results showed that the fracture toughness of the graphene/alumina nanocomposites increased by 53% with the addition of 2 wt.% GNS compared to monolithic alumina. Furthermore, the GNS-reinforced Al_2_O_3_ composites resulted in the refinement of the alumina grain size. This may be attributed to the nanosheets inhibiting the growth of the grain during sintering. Porwal et al. [25] fabricated graphene nanoflake (GNF)-reinforced alumina nanocomposites using a powder metallurgy technique. In their study, 0.2 wt.%, 0.5 wt.%, 0.8 wt.%, 2 wt.%, and 5 wt.% GNF/Al_2_O_3_ specimens were prepared by a liquid phase exfoliation method and then consolidated using the SPS technique. They reported that the fracture toughness of the GNF/alumina nanocomposites increased by 40% with the addition of only 0.8 wt.% GNF. Chen et al. [26] produced GNP/Al_2_O_3_ composites with 0.1 wt.%, 0.2 wt.%, 0.5 wt.%, and 1 wt.% GNP contents utilizing the hot pressing process. Their results indicated that the addition of GNPs improved the fracture toughness of the composites by 43.5% higher than that of the monolithic alumina. Liu et al. [27] produced GNS/Al_2_O_3_ with 0.1 vol.%, 0.3 vol.%, 0.6 vol.%, 2.0 vol.%, and 3.5 vol.% contents using the ball-milling method and spark plasma sintering. They reported that the GNS/Al_2_O_3_ nanocomposites exhibited higher fracture toughness, flexural strength, and Vickers hardness by approximately 25%, 103%, and 26%, respectively, compared to monolithic alumina. Ahmad et al. [10] fabricated highly dense graphene nanosheet-reinforced alumina nanocomposites using a high-frequency induction heating system (HFIHS). They studied the effects of the GNS on the nanocomposites’ hardness, elastic modulus, fracture toughness, and microstructure. The obtained results showed that the addition of 0.5 wt.% GNS contents significantly improved the hardness and fracture toughness compared to the other samples. Kim et al. [28] fabricated GNP/Al_2_O_3_ nanocomposites using the high-energy ball-milling process and high-frequency induction heated sintering. They reported that the addition of 1 wt.% and 3 wt.% graphene contents to alumina led to significant improvement in the hardness and fracture toughness of the fabricated composite. Liu et al. [29] used the ball-milling process and a pressureless furnace to fabricate Al_2_O_3_ nanocomposites reinforced with 0.75 vol.%, 1.3 vol.%, and 1.48 vol.% GNP contents. They reported that the 0.75 vol.% GNP content-reinforced Al_2_O_3_ nanocomposites had significantly improved flexural strength and fracture toughness by approximately 60% and 70%, respectively. Ahmad et al. [1] studied the influence of multi-layer graphene (MLG) contents (0.5 vol.% and 1.00 vol.%) on the density, hardness, structural, wear resistance, and tribological properties of fabricated nanocomposites using an HFIHS. The results showed that the nanocomposites with 1 vol.% MLGs exhibited 15% and 25% lower friction coefficients than the nanocomposites with 0.5 vol.% MLGs and monolithic Al_2_O_3_, respectively. Ahmad et al. [30] studied the effect of adding GNPs on the thermophysical properties of fabricated GNP/Al_2_O_3_ nanocomposites using HFIH. The obtained results showed that the nanocomposites with 0.5 wt.% GNP content exhibited hardness and fracture toughness values of 18.4 GPa and 5.7 Joule/m, respectively, which were higher than those of monolithic Al_2_O_3_. Moreover, the Al_2_O_3_ showed the highest thermal conductivity value. Shah et al. [11] studied the effects of graphene content (0 wt.%, 0.4 wt.%, 0.8 wt.%, 1.2 wt.%, and 1.6 wt.%) on the density, microstructure, fracture toughness hardness, and strength of graphene-reinforced Al_2_O_3_ nanocomposites. The nanocomposite samples were prepared by ultra-sonication and the SPS process. The results showed that the density and bending strength slightly decreased with increasing graphene content from 0.4 wt.% to 1.2 wt.%. Moreover, all the nanocomposites revealed improved fracture toughness compared to the monolithic Al_2_O_3_ samples. Table 1 summarizes some of the works reported on the preparation, characterization, and machining of Al_2_O_3_ nanocomposites reinforced with graphene.

As is evident in the literature, adding graphene-based reinforcements has a great impact on the physical and mechanical properties of Al_2_O_3_. Therefore, there is immense potential in exploring their machining behavior. Several studies have been reported in the literature regarding the machining of pure Al_2_O_3_ ceramic [36,37], but studies related to the machining of graphene-based alumina nanocomposites are scarce. As shown in Table 1, only Sung et al. [38] investigated the electrical discharge machining (EDM) of the graphene-reinforced Al_2_O_3_ nanocomposites, while the remaining studies only focused on their characterization. Sung et al. [38] reported that an increase in the electrical conductivity of the graphene-reinforced Al_2_O_3_ nanocomposites led to significant improvement in their EDM. Moreover, they found that an increase in the surface roughness was observed at the high graphene content of 15 wt.%.

No detailed work has been presented so far on the effect of graphene reinforcement on the machining behavior of these new materials. Only a study reported by Lee et al. [38] explored the effect of GNPs and CNT on optical absorbance and thermal conductivity. They found that the optical absorbance and thermal conductivity of nanocomposites were improved compared to pure alumina. To utilize graphene-reinforced Al_2_O_3_-based nanocomposites in various applications, it is essential to study their machinability in detail. Therefore, this study aimed to develop a detailed understanding of the effect of graphene reinforcement to improve the microfabrication performance of Al_2_O_3_-based nanocomposites to satisfy sustainable manufacturing requirements. To achieve this aim, high-density Al_2_O_3_ nanocomposite samples were produced with different graphene contents using dry ball-milling and HFIHS techniques. The density, hardness, and microstructure were studied to evaluate the nanocomposite specimens. After that, detailed micromachining experiments were conducted to study the effect of the GNP contents and machining parameters on the microfabrication performance of Al_2_O_3_-based nanocomposites. The surface integrity, surface roughness, ablation depth, and material removal rate were used to compare the microfabrication performance of the developed GNP/Al_2_O_3_ nanocomposites.

## 2. Experimental Procedure

### 2.1. Fabrication of Nanocomposites

**Figure 1:**
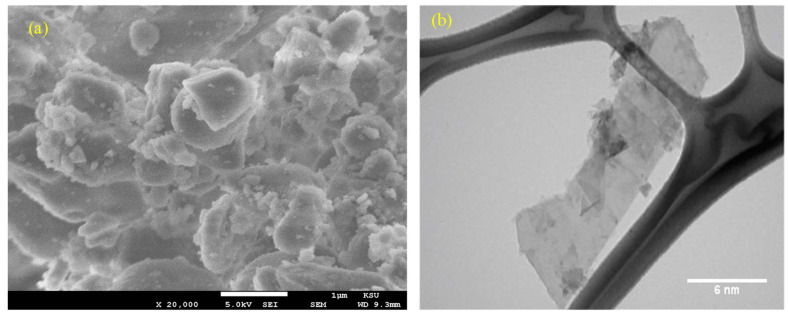
Morphology of the received powder. (**a**) Al_2_O_3_ and GNPs; (**b**) GNPs (XG Sciences, Inc., USA).

Commercial alumina powder with a particle size of 300 nm was used as the matrix material, supplied by US Research Nanomaterials, Inc. (Houston, TX, USA). The chemical composition of the received alumina powder is provided in Table 2. Figure 1a shows the morphology of the received Al_2_O_3_ powder.

Graphene nanoplatelets (GNPs), supplied by XG Sciences, Inc. (Lansing, MI, USA), were used as the reinforcement material. The characteristics of the GNPs are listed in Table 3. The morphology of the received GNP powder is shown in Figure 1b.

**Figure 2:**
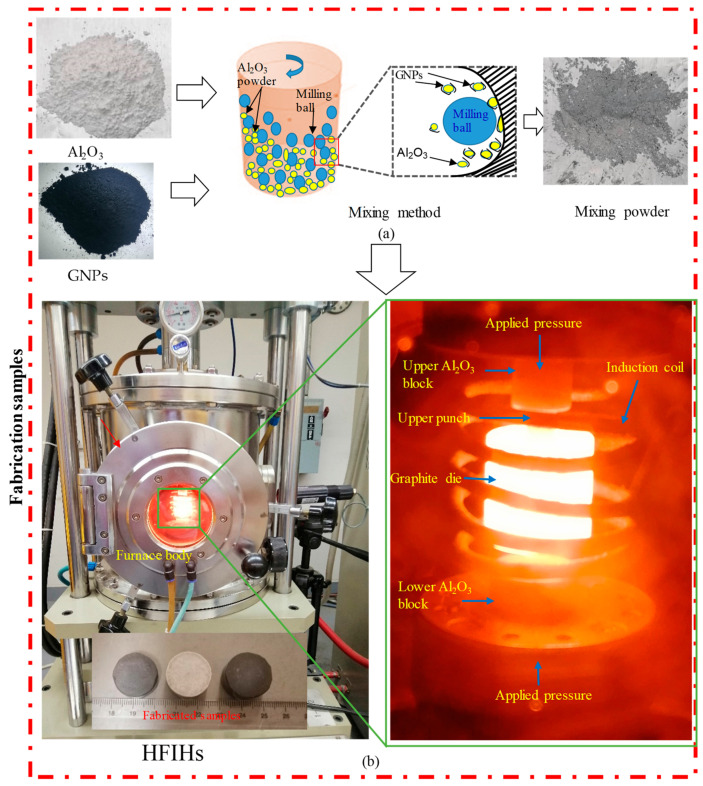
Experimental setup for fabricating the GNP/Al_2_O_3_ specimens.

To fabricate the GNP-reinforced Al_2_O_3_ nanocomposites, the initial powder mixing was a critical step in ensuring the homogenous dispersion of the GNPs in the base Al_2_O_3_ powder. Several techniques mentioned in Table 1 are used for powder mixing. In this work, the planetary ball-milling technique was used to prepare the nanocomposites. All the powders (with or without GNPs) were mechanically ball-milled using the Pulverisette machine (FRITSCH GmbH, Idar-Oberstein, Germany). The GNP/Al_2_O_3_ nanocomposites were prepared using different weight percentages of reinforcement, including 0 wt.% (base Al_2_O_3_), 0.5 wt.%, 1 wt.%, 1.5 wt.%, and 2.5 wt.% GNPs. The GNP weight percentages were selected based on the preliminary experiments, which demonstrated that using a GNP wt.% of >2.5 led to compromised mechanical properties. The ball-milling was performed in cylindrical zirconia containers using yttria-stabilized zirconia balls (diameter = 15 mm) at 350 rpm for 4 h under a ball-to-powder weight ratio of 20:1 [39]. A schematic diagram of the ball-milling process of GNP-reinforced nanocomposites is illustrated in Figure 2a. The ball-milled powder was loaded into a graphite die with an internal diameter of 20 mm and then consolidated by an HFIHS furnace (HF Active Sinter System, ELTek CO., Gyeonggi-do, Republic of Korea), as shown in Figure 2b, at a temperature of 1500 °C, a heating rate of 150 °C/min, uniaxial pressure of 60 MPa, and a cooling rate of 200 °C/min in a vacuum (45-Torr) [10,30]. The dimensions of the fabricated specimens were 20 mm in diameter and 12 mm in height.

### 2.2. Machining and Measurements Setups

**Figure 3:**
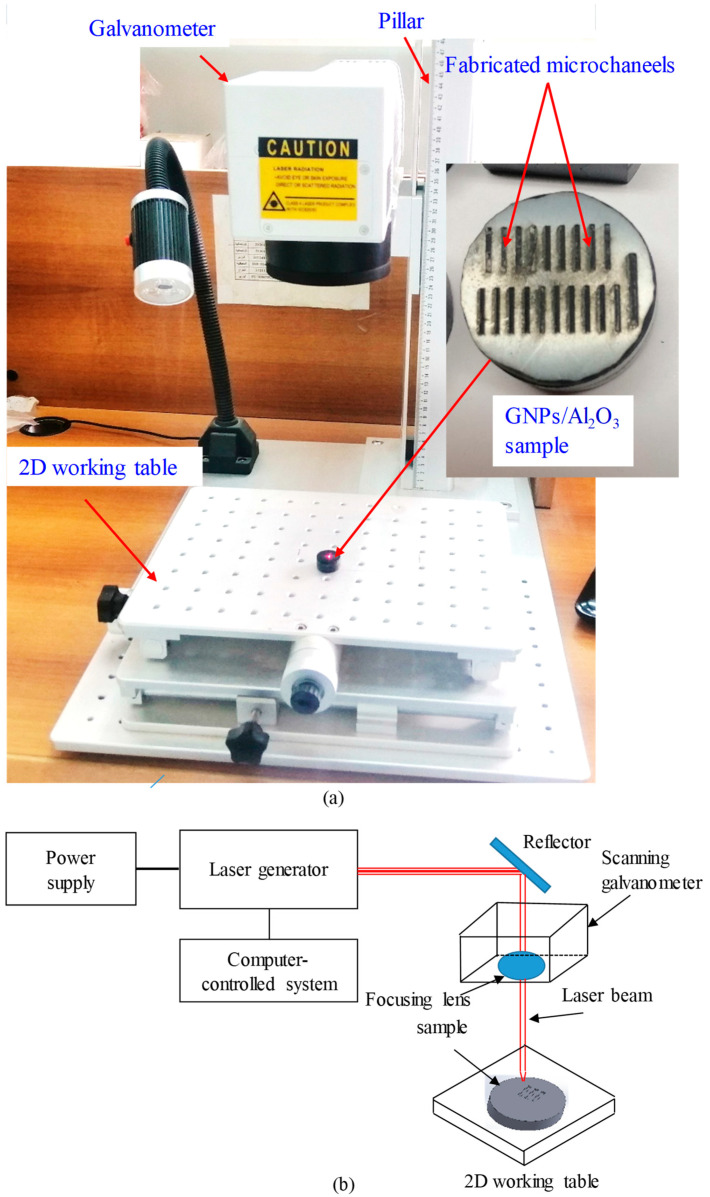
(**a**) Laser beam machining setup; (**b**) schematic diagram of laser beam machining.

Laser microfabrication experiments were performed on the GNP/Al_2_O_3_ nanocomposites using an XTL-FP 20 laser machine from XT laser, Jinan, China, as shown in Figure 3a. This fiber laser requires very low power consumption (20 W). The laser beam was focused using a flat-field lens, moved through a galvanometric mirror system, and irradiated on the top surface of the fabricated GNP/Al_2_O_3_ specimens. Figure 3b shows a schematic diagram of laser beam machining. These types of laser sources have been designed specifically for the marking/engraving of metallic materials, such as steel and aluminum. In addition, they are adopted for different applications, such as surface treatments and micromachining operations. However, in this work, this low-power and low-cost laser machine was adapted for testing the machinability of the GNP/Al_2_O_3_ ceramic nanocomposites. It is worth mentioning that pure Al_2_O_3_ ceramic is extremely difficult to machine even with sophisticated high-power laser machines, such as that reported by [40]. Poor machining in terms of the microchannel shape and dimensions and the surface integrity have been reported even using expensive laser setups [40,41]. In this paper, it is shown that the addition of GNPs in Al_2_O_3_ enabled high-quality laser machining even with a very low cost, low power (20 W), and an easily accessible laser machine (XTL-FP 20 laser). This meets the requirements of clean, sustainable manufacturing where high-quality products (e.g., microchannels) can be produced with lower costs and resources.

Microchannels with the dimensions of 250 µm in width and 5 mm in length were fabricated on all produced GNP/Al_2_O_3_ nanocomposites with varying GNP contents. Before the main experiments, all fabricated samples were ground to remove the superficial graphite and oxide layers and non-uniformity. Later, two laser parameters, i.e., the scanning speed and pulse frequency, were varied during the laser micro-milling. There is no information available in the literature regarding the laser micromachining of GNP/Al_2_O_3_ matrix nanocomposites. Therefore, initially, preliminary tests were conducted to identify the suitable ranges of influential factors using the reported studies on the micromachining of alumina ceramics [40,42,43]. Table 4 shows the selected laser parameters and their ranges.

**Figure 4:**
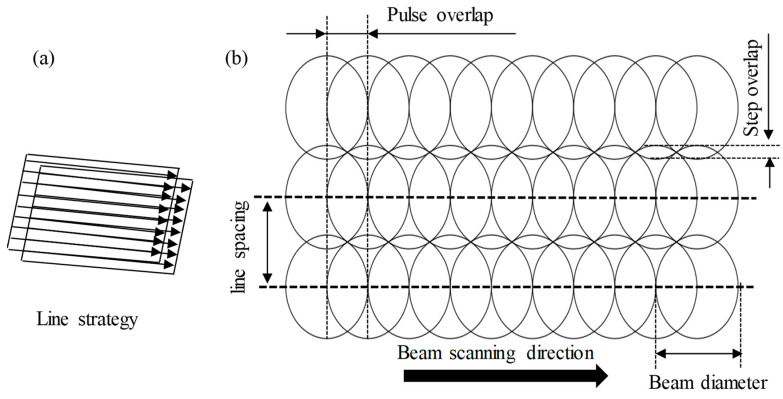
(**a**) Laser scanning strategy; (**b**) schematic of the adjacent laser tracks.

**Figure 5:**
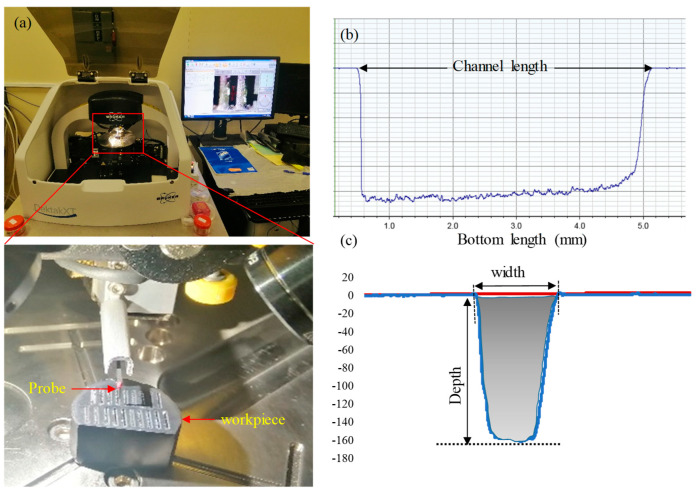
Measuring devices. (**a**) Three-dimensional optical profilometer; (**b**,**c**) typical geometry of the 2D profile and its cross-sectional area.

Parameters such as the power (20 W), scanning strategy (line strategy), and line spacing were kept constant throughout all the tests, as shown in Figure 4. In addition, to evaluate the effects of graphene content on the fabrication of microchannels, five specimens with 0.0 wt.%, 0.5 wt.%, 1 wt.%, 1.5 wt.%, and 2.5 wt.% GNPs were machined with the same laser parameters. Each test was repeated three times for each microchannel to guarantee the repeatability of the experiments, and later, the average value was used. The output responses, including the microchannels’ geometries (depth and top width), material removal rate, surface roughness, and surface morphology, were used to appraise the influence of the graphene contents on the micromachining behavior of the GNP/Al_2_O_3_ nanocomposites. After machining, all machined specimens were first cleaned using ethanol to remove any loose debris or contaminants on the fabricated microchannels [44]. Then, the dimensional accuracy and surface roughness were measured by a 3D optical profilometer (DektakXT Stylus Profiler) from Bruker (Billerica, MA, USA). The optical 3D profilometer was equipped with an inductive gauge of 12.5 μm radius diamond stylus, as shown in Figure 5a. The ablated depth and width were measured by capturing four random 2D profiles along the width of the fabricated channels with a scanning speed of 5 μm/s (see Figure 5c). The average of the measured values was used later to measure the dimensional accuracy (depth and width). The roughness was measured in terms of the arithmetic mean surface roughness (Ra) according to the ISO 4287 standard. The surface roughness was measured by scanning four random measurements along the bottom length of the channels, and averaged values were used for further analysis (see Figure 5b). The surface morphologies of the machined microchannels were analyzed using a scanning electron microscope (SEM) from Jeol Japan (Model JCM 6000 Plus). Before capturing the SEM images, a platinum coating was applied to all machined samples using the JFC 1600 auto fine coater (JEOL Ltd., Tokyo, Japan) to enhance their visibility. The material removal rate (MRR) was used to assess the effect of the graphene contents on the laser micromachining of the nanocomposites. The MRR was calculated using Equation (1).

(1) $$\mathrm{MRR}=\frac{\mathrm{Machined}\;\mathrm{area}\times \;\mathrm{machined}\;\mathrm{length}}{\mathrm{machining}\;\mathrm{time}}\;\left({\mathrm{mm}}^{3}/\min\right)\;$$

The cross-section of the machined area was calculated from the fitted 2D profile, as shown by the grey-filled area in Figure 5c. The cross-sectional area of the four profiles was averaged and then used in Equation (1).

## 3. Results and Discussions

### 3.1. Characterization of the Fabricated GNP/Al_2_O_3_-Based Nanocomposites

#### 3.1.1. Microstructure Evaluation

**Figure 6:**
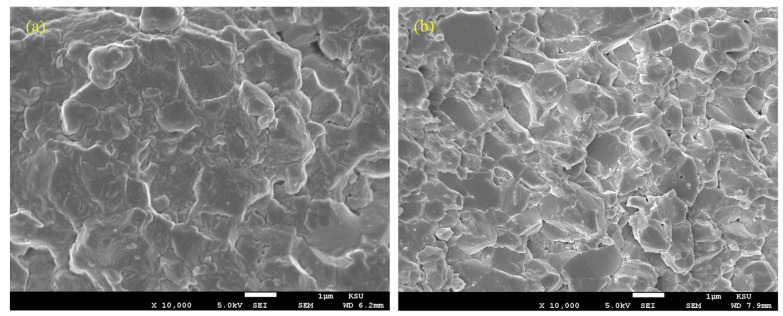
SEM images showing the fractured surfaces of (**a**) base Al_2_O_3_; (**b**) 1.5 wt.% GNP/Al_2_O_3_.

Figure 6 shows the morphologies of the fractured surfaces for the base Al_2_O_3_ and the GNP/Al_2_O_3_ nanocomposites. It can be seen that the GNP/Al_2_O_3_ nanocomposites had a smaller grain size compared to the base Al_2_O_3_. The addition of the GNPs tended to restrict grain growth by acting as an obstruction between the Al_2_O_3_ matrix particles.

#### 3.1.2. Density Analysis

**Figure 7:**
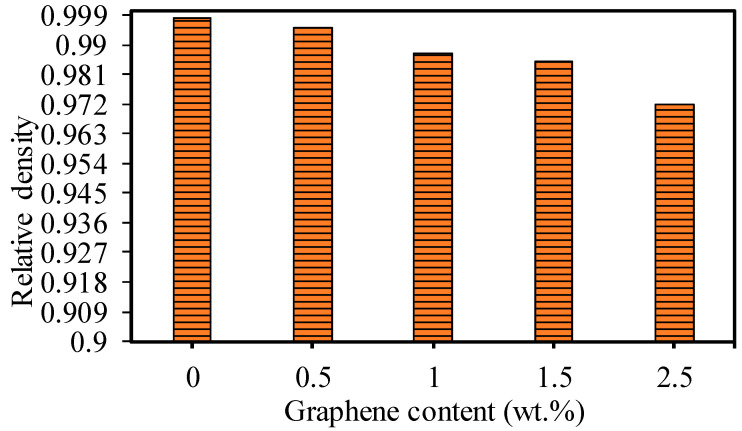
Relative density of the base Al_2_O_3_ and GNP/Al_2_O_3_ nanocomposites with different GNP contents.

The actual density of the fabricated GNP/Al_2_O_3_ nanocomposites was measured using the Archimedes method by employing the density measurement system from Sartorius Lab Instruments, Goettingen, Germany. Afterward, the relative density was calculated by dividing the actual density by the theoretical density [45] of the powder mixture, as shown in Figure 7. It can be seen in Figure 7 that the fabricated GNP-reinforced Al_2_O_3_ nanocomposite samples exhibited a high relative density, which indicates good bonding between the GNPs and the Al_2_O_3_ matrix, with negligible porosity or cavities.

#### 3.1.3. Hardness

**Figure 8:**
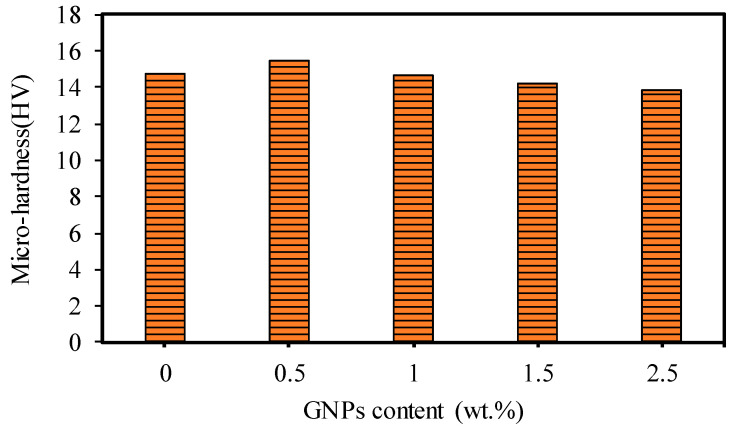
Hardness for the base Al_2_O_3_ and GNP/Al_2_O_3_ nanocomposites.

The ZHV30 Vickers Hardness Tester was used to measure the Vickers hardness of the fabricated specimens using a load of 30 kg for a dwell time of 12 s. For each specimen, the hardness measurement was repeated six times at different locations on the ground surface, and later, the average value was used, as shown in Figure 8. It can be seen that nanocomposites with 0.5 wt.% GNPs had the highest hardness by approximately 7% compared to the base Al_2_O_3_. This can be attributed to the presence of GNPs among the Al_2_O_3_ particles, inhibiting grain growth and resulting in a smaller grain size and good interfacial bonding between the Al_2_O_3_ particles [10,16]. With GNP contents of 1 wt.% and 1.5 wt.%, the Vickers hardness slightly decreased. This can be attributed to the presence of the thicker layer of GNPs among the Al_2_O_3_ grains, which led to a weakening of the interfacial bonding between the Al_2_O_3_ particles and reduced hardness [1,10,16].

### 3.2. Micromachining Results and Discussion

#### 3.2.1. Surface Morphology

**Figure 9:**
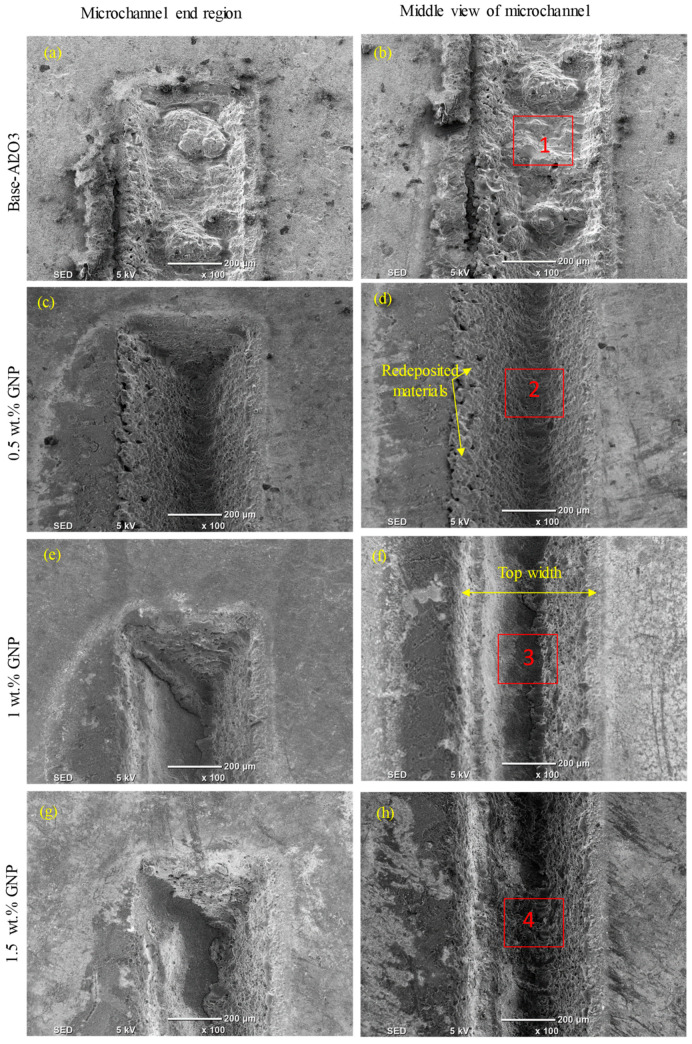

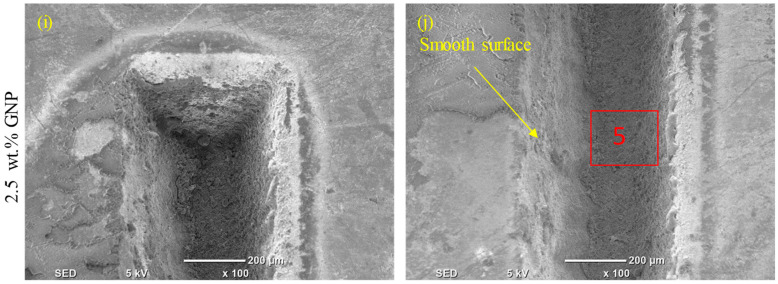
Surface morphology of the ablated base Al_2_O_3_ and GNP/Al_2_O_3_ nanocomposites with varying graphene contents at 200 mm/s.

**Figure 10:**
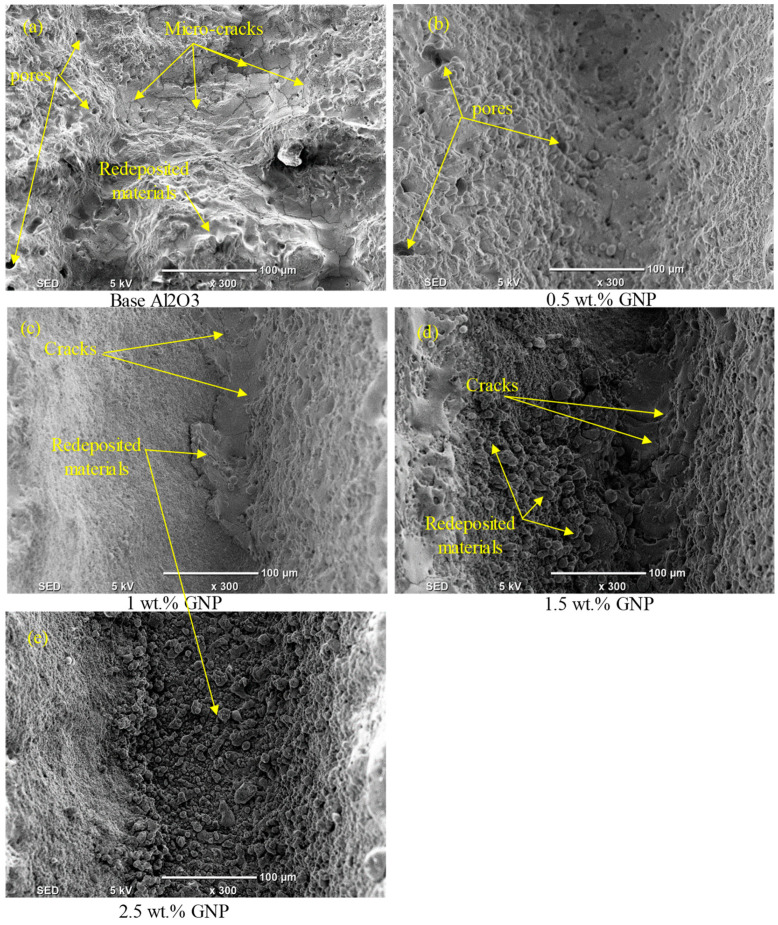
Zoomed-in images of the selected regions (1–5) shown in Figure 9.

**Figure 11:**
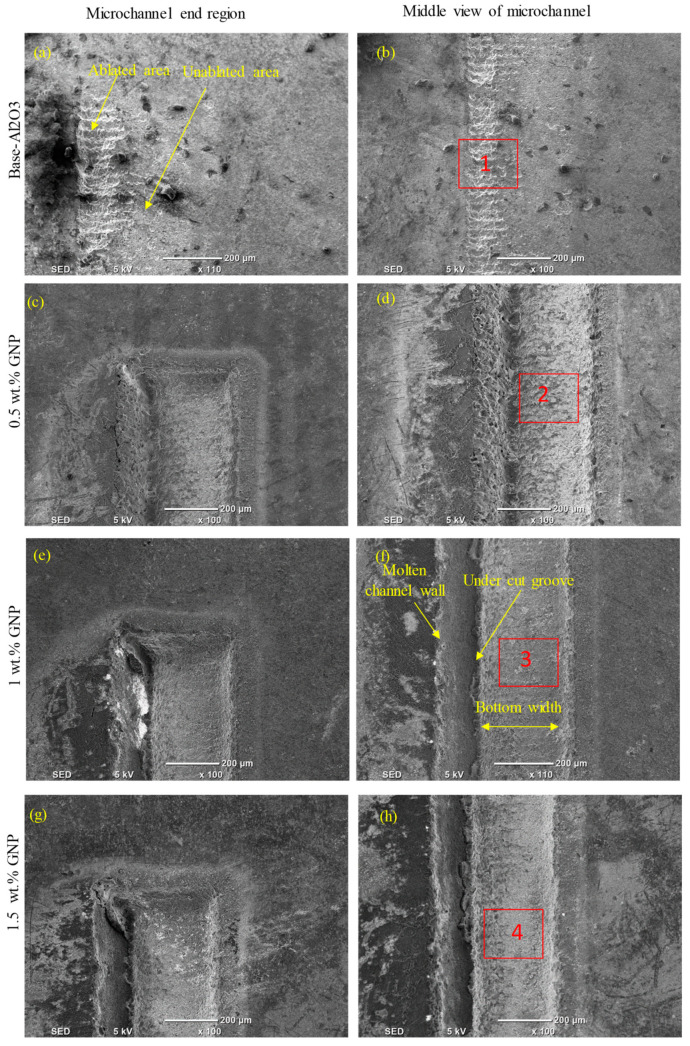

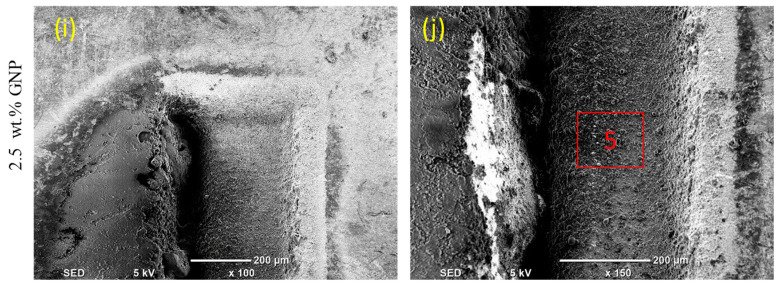
Surface morphologies of the ablated base Al_2_O_3_ and GNP/Al_2_O_3_ nanocomposites with varying graphene contents at 500 mm/s.

**Figure 12:**
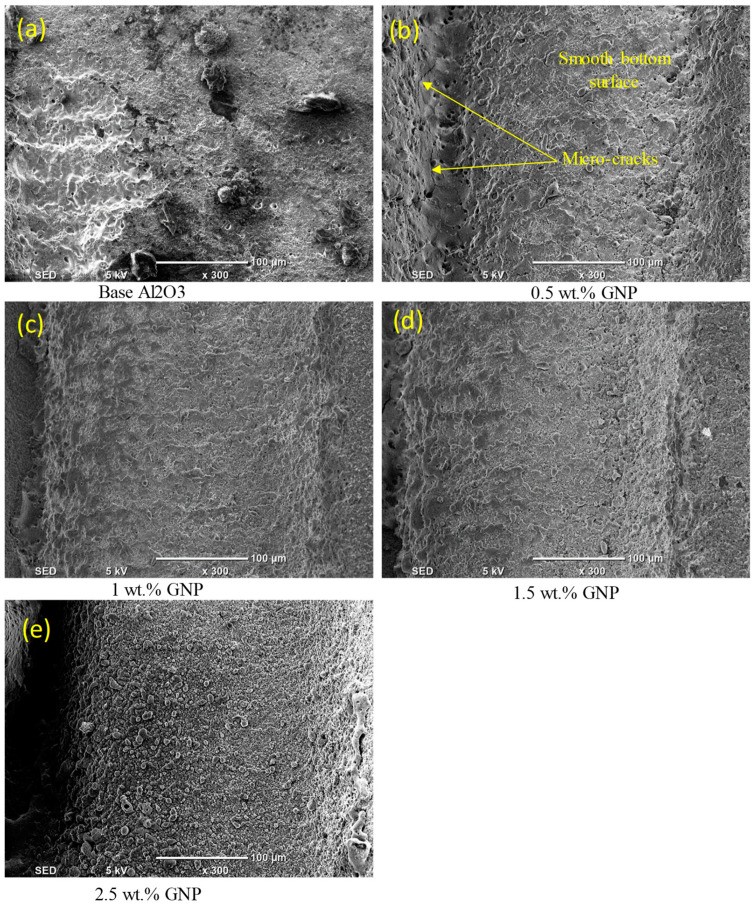
Zoomed-in images of the selected regions (1–5) shown in Figure 11.

Figure 9, Figure 10, Figure 11 and Figure 12 show the surface morphologies of the laser-fabricated microchannels on the base Al_2_O_3_ and GNP/Al_2_O_3_ nanocomposites at lower (200 mm/s) and higher (500 mm/s) scan speeds. The results show that the microchannels of the GNP/Al_2_O_3_ nanocomposites had overall better surface quality and geometry compared to the base Al_2_O_3_ specimens in all machining conditions. This is because of the presence of the GNPs among the Al_2_O_3_ matrix particles, which enhanced the thermal conductivity and optical absorbance properties during the laser micromachining. These properties induced higher surface melting and evaporation at even lower energy densities. Kim et al. [46] found that the presence of CNT enhanced the machinability of CNT/Fe/Al_2_O_3_ nanocomposites due to the addition of the CNT, which led to lower light transmittance, higher thermal conductivity, and suppressed grain growth.

At a scan speed of 200 mm/s (see Figure 9), it can be seen that redeposited material, cracks, and pores were formed on the bottom surface of the microchannel and on the surrounding sidewalls in the case of the base Al_2_O_3_, as shown in Figure 9a,b and Figure 10a. On the contrary, a much more regular and smoother channel was observed for the nanocomposites with 0.5 wt.% GNPs at a scan speed of 200 mm/s, as can be seen in Figure 9c,d and Figure 10b. The surface morphology of the ablated microchannels shows a higher ablation with few cracks and pores on the bottom and sidewalls of the microchannels (see Figure 9c,d and Figure 10b). This can be attributed to the higher energy absorption of the nanocomposites, which required less energy for melting the surfaces of the specimens. For the 1 wt.% GNP specimens, the fabricated microchannels on the nanocomposites had visible and irregular redeposited materials and microcracks on the bottom surface at a scanning speed of 200 mm/s. In addition, the sidewalls of the microchannels became smoother with a marginal recast layer compared to the specimens with a 0.5 wt.% GNP content (see Figure 9e,f and Figure 10c). This was due to the increase in the GNP contents, which increased the optical absorbance of the specimens, causing more melting materials compared to the 0.5 wt.% GNP specimens. On the contrary, for the 1.5 wt.% GNP/Al_2_O_3_ nanocomposite, the surface morphology of the fabricated channel had a rough surface at a lower scanning speed of 200 mm/s compared to the 0.5 wt.% and 1 wt.% GNP specimens (see Figure 9g,h and Figure 10d). This was because of the lower ablation threshold energy for the 1 wt.% GNP specimens, which induced more melting material compared to the lower GNPs content. In addition, the increase in the GNP content resulted in an increase in the material’s thermal conductivity, which allowed the molten material at the bottom and sides of the microchannels to resolidify faster. This was observed as thicker re-solidified layers, microcracks, and pores around the side wall, and rougher bottom surfaces of the microchannels as the GNP wt.% increased. For the same reasons, the nanocomposites with 2.5 wt.% GNPs exhibited the worst surface morphology, with more redeposited material and pores compared to the 0.5 wt.%, 1 wt.%, and 1.5 wt.% GNP specimens, as shown in Figure 9i,j and Figure 10e.

At a high scanning speed of 500 mm/s, it was evident that no well-defined microchannels were created; instead, only multiple-pulse traces of the laser beam were seen on the base Al_2_O_3_ specimens. The base Al_2_O_3_ presented poor laser machinability compared to all of the GNP/Al_2_O_3_ nanocomposites, as shown in Figure 11a,b and Figure 12a. The laser-ablated nanocomposites with 0.5 wt.% GNPs showed well-defined microchannels. In addition, the ablated depths of the microchannels decreased, and some cracks, pores, and redeposited materials on the bottoms of the channels were formed, resulting in an increase in the surface roughness (see Figure 11c,d and Figure 12b). Moreover, it can be seen in the SEM images that the microchannel shape changed from triangular to trapezoidal with the increase in the scanning speed from 200 mm/s to 500 mm/s (compare Figure 9c and Figure 11c). For the 1 wt.%, 1.5 wt.%, and 2.5 wt.% GNP specimens, smoother surface morphologies were noted at 500 m/s compared to the 0.5 wt.% GNP specimens (see Figure 11e–j and Figure 12c–e), contrary to the scanning speed at 200 mm/s. This is because at a higher scanning speed (500 mm/s), the laser–workpiece interaction time was reduced, and samples with a higher ablation threshold (0.5 wt.% GNPs) were not adequately irradiated to melting and evaporation. As the GNP contents increased, the optical absorbance of the specimens increased, resulting in more molten and evaporated material from the walls and bottoms of the channels compared to the 0.5 wt.% GNP specimens (Figure 11i,j and Figure 12e).

#### 3.2.2. Microchannel Accuracy

**Figure 13:**
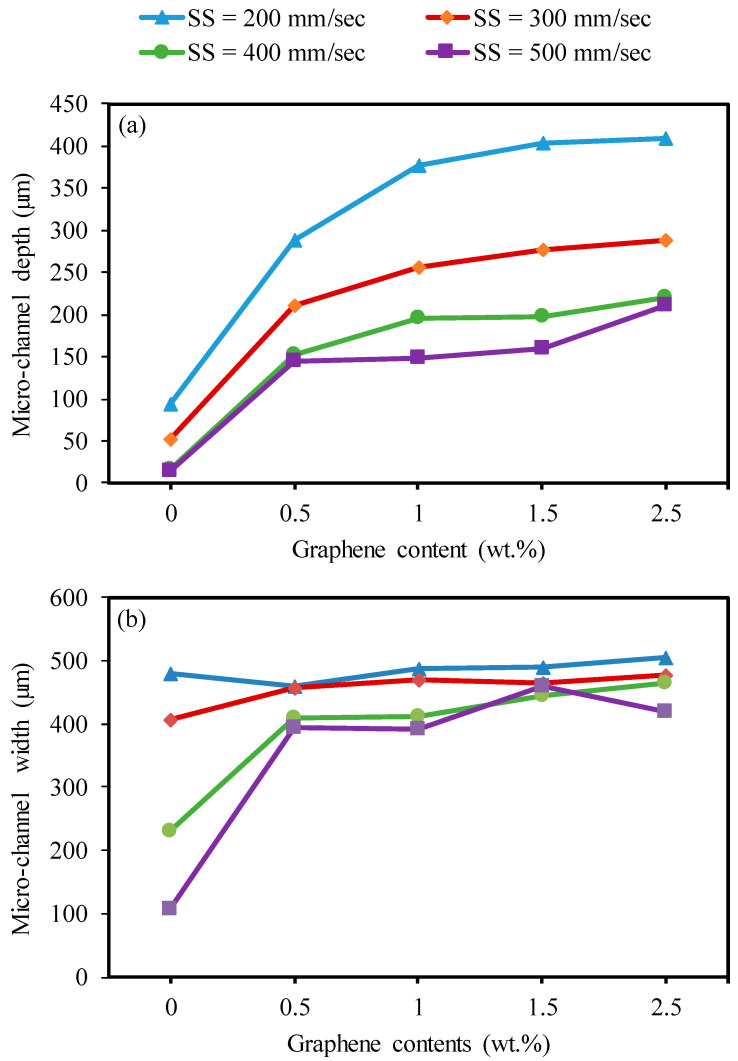
Effects of varying graphene reinforcement contents with laser speed on microchannel depth and width for machining base Al_2_O_3_, and GNP/Al_2_O_3_ nanocomposites at F = 30 kHz. (**a**) Microchannel depth, (**b**) Microchannel depth.

Figure 13a,b shows the effect of the GNP contents with varying scanning speeds on the channel depth and width. It can be concluded from Figure 13a that the channel depth increased with an increment in the GNP percentage from 0.0 wt.% to 2.5 wt.%. This is attributed to the improved thermal conductivity and optical absorbance of the GNP/Al_2_O_3_ nanocomposites, as discussed in Section 3.2.1 [31,39,47], leading to lowering the ablation threshold of the composites due to the addition of the GNPs. Therefore, the channel depth increased for the GNP/Al_2_O_3_ nanocomposites by around two to nine times compared to the base Al_2_O_3_. In addition, the channel depth increased with the increasing graphene contents from 0.5 wt.% to 2.5 wt.% due to further increases in the thermal conductivity, optical absorbance, and reduced grain size [48].

**Figure 14:**
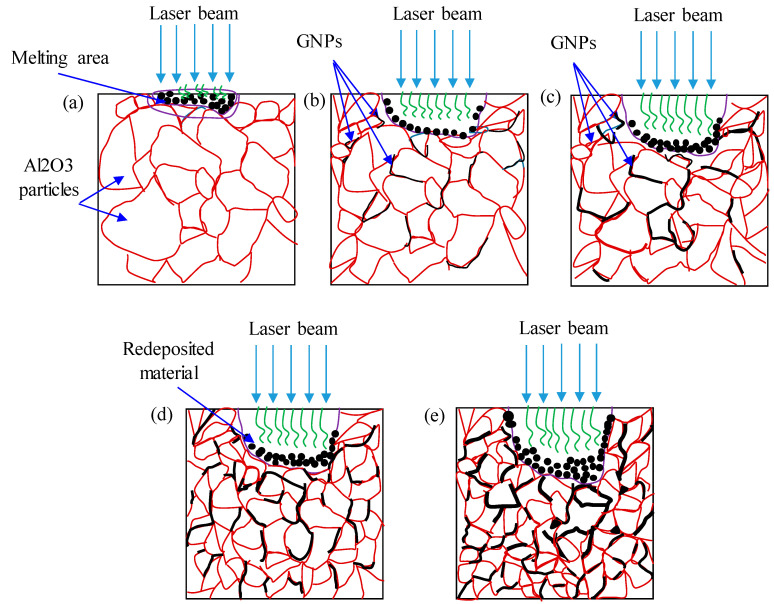
Schematic diagram of laser beam machining, (**a**) base Al_2_O_3_; (**b**) 0.5 GNP/Al_2_O_3_ nanocomposites; (**c**) 1 wt.% GNP/Al_2_O_3_ nanocomposites; (**d**) 1.5 wt.% GNP/Al_2_O_3_; (**e**) 2.5 wt.% GNP/Al_2_O_3_ nanocomposites.

At a higher scanning speed of 500 mm/s, the channel depth tended to decrease slightly with the GNP content rising from 1 wt.% to 1.5 wt.%, and then increased with the GNP content rising to 1.5 wt.%. The main reason for the improvement in the microchannel depth is shown in Figure 14. When the graphene content increased from 0.0 wt.% to 0.5 wt.%, more material melted, and less material was redeposited on the microchannel bed. Hence, the microchannel depth increased (see Figure 14b). When the graphene content increased from 0.5 wt.% to 1 wt.%, the microchannel depth increased because more induced materials were ablated, and few materials were redeposited on the microchannel bed, as can be seen by comparing Figure 14b,c. When the GNPs increased to 2.5 wt.%, the ablation threshold energy was reduced, which led to melting and more material redeposition on the machined surface. This was because there was a significant amount of thick graphene surrounding the Al_2_O_3_ particles (see Figure 14d,e) which efficiently transferred the heat away from the melting zone, causing redeposited materials on the beds and the sidewalls of the channels. Hence, these redeposited materials slightly increased the channel depth.

Figure 13b shows the impact of the graphene content with different scanning speeds on the microchannel widths of all fabricated samples. It can be seen that the results of the channel widths of the GNP/Al_2_O_3_ nanocomposites are similar in most of the machining conditions. However, some points could be noted. In the cases of the 1.5 wt.% and 2.5 wt.% GNP specimens, the width increased as the GNP content increased from 1 wt.% to 2.5 wt.% at a scanning speed of 400 mm/s. These results can be explained as follows. The absorption of the laser energy in the GNP nanocomposites increased with the increasing graphene content, which affected the melting process and the vaporization [38]. Therefore, at low scanning speeds of 200 mm/s and 300 mm/s, there was more interaction time between the laser and materials, leading to more molten material and rapid evaporation, which resulted in increased depths and stabilization of the widths, without any changes in any of the GNP/Al_2_O_3_ nanocomposites [49]. In the case of the high scanning speeds of 400 mm/s and 500 mm/s, it was expected that the nanocomposites with 1.5 wt.% and 2.5 wt.% GNPs would have a high absorption of energy compared to those with 0.5 wt.% and 1 wt.% GNPs, which resulted in more molten and evaporated materials from the walls and bottoms of the channels, leading to an increased width (see Figure 11a–j. However, in the case of the 2.5 wt.% GNP specimens, the width decreased at a scanning speed of 500 mm/s. This was because there was a significant amount of thick graphene surrounding the Al_2_O_3_ particles (see Figure 14e), which efficiently transferred the heat away from the melting zone, causing redeposited materials on the sidewalls of the channels. Hence, these redeposited materials reduced the channel width (as can clearly be seen in Figure 12e. In the case of the pure Al_2_O_3_, the width decreased as the scanning speed decreased. This happened because of the decrease in the absorption of energy, which led to a reduction in the melting and evaporation, resulting in a decreased width (see Figure 9a–i).

**Figure 15:**
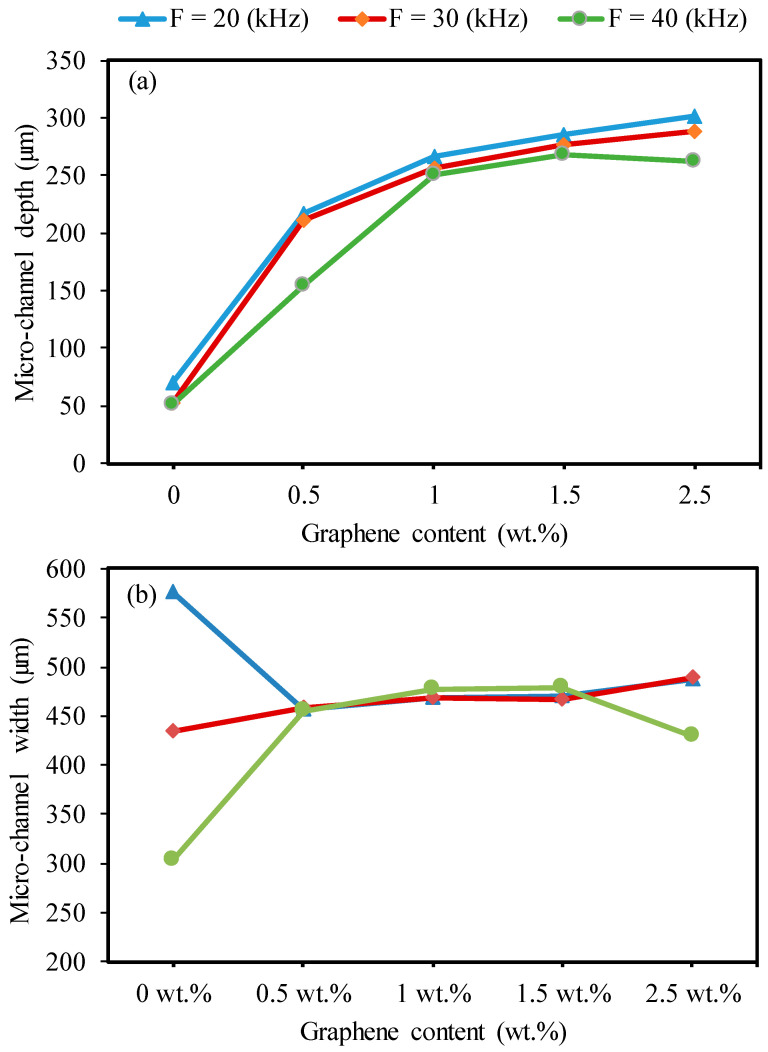
Effects of the graphene reinforcements and frequency on the microchannel depth for machining base Al_2_O_3_ and GNP/Al_2_O_3_ nanocomposites (SS = 300 mm/s). (**a**) Microchannel depth, (**b**) Microchannel depth.

Figure 15 shows the trends of the impact of graphene contents on the microchannel depth and width at different levels of frequency. Regarding the effect of the GNPs, it can be concluded from Figure 15a that the nanocomposites reinforced with different graphene contents showed improvement in the channel depth compared to the base Al_2_O_3_. This trend was nearly the same as in the case of Figure 13. That is, the channel depth increased with increasing graphene contents from 0.5 wt.% to 2.5 wt.%. This is attributed to the optical absorbance and ablation threshold of the GNP/Al_2_O_3_ nanocomposites, which can be explained as follows. The pulse energy and pulse power identify the energy density and power density of the laser beam–material interaction mode and, therefore, the amount of machined volume. Leone et al. [41] found that the energy density was reduced with an increasing pulse frequency. The differences in the channel depth for the 20 kHz results in comparison to the higher frequencies can be seen as an indication of these changes. The presence of graphene among the alumina particles led to enhanced thermal conductivity and optical absorbance of the nanocomposites. However, these nanocomposites required a lower ablation threshold pulse energy for melting the materials compared to the base alumina. With an increasing frequency from 30 to 40 kHz, less material was molten during the ablation of the Al_2_O_3_ because the increasing frequency generated less energy. These results are in line with a reported study on the laser machining of Al_2_O_3_ [41]. However, in the cases of the 0.5 wt.%, 1 wt.%, 1.5 wt.%, and 2.5 wt.% GNP specimens, the graphene enhanced the effects of the increasing frequency on generating a lower energy density. Therefore, more material was ablated and removed from the bottom of the channel compared to the base Al_2_O_3_. However, an increased channel depth was obtained by the nanocomposites with 2.5 wt.% GNP contents. Kim et al. [46] reported that Al_2_O_3_ nanocomposites with higher CNT contents exhibited a higher ablation rate compared to those with low CNT contents due to lower light transmittance, higher thermal conductivity, and a smaller grain size. In the case of the 2.5 wt.% GNP specimens, the depth and width decreased at a frequency of 40 kHz due to the thick graphene, which conducted the heat away from the melting zone, leading to redeposited material on the wall and bottom (see Figure 14e).

Regarding the effect of graphene content on the width, as shown in Figure 15b, the results are similar, and no obvious differences were observed in the width among the 0.5 wt.%, 1 wt.%, 1.5 wt.%, 2.5 wt.% GNP specimens. In the case of the base Al_2_O_3_, the width sharply decreased with increasing frequency due to less energy absorbance, as explained earlier.

**Figure 16:**
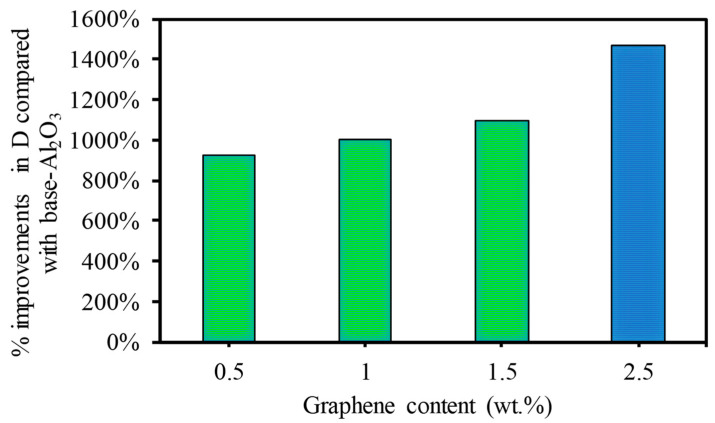
Percentage of improvement in ablation depth at a higher scanning speed of 500 mm/s.

To show the benefits of the GNP content on the laser machining of Al_2_O_3_, Figure 16 is provided, which shows the improvement in the ablation depth of the GNP/Al_2_O_3_ nanocomposites compared to the base Al_2_O_3_. It can be observed that the addition of GNPs enhanced the laser micromachining by increasing the ablation depth at lower and higher scanning speeds. For instance, at higher scanning speeds, the ablation depth was increased by 982%, 1004%, 1094%, and 1467% for the 0.5 wt.%, 1 wt.%, 1.5 wt.%, and 2.5 wt.% GNP/Al_2_O_3_ nanocomposites as compared to the base Al_2_O_3_ (see Figure 14). The results of the 2.5 wt.% GNP reinforcement exhibited the highest ablation depth during the laser beam micromachining.

#### 3.2.3. Surface Roughness

**Figure 17:**
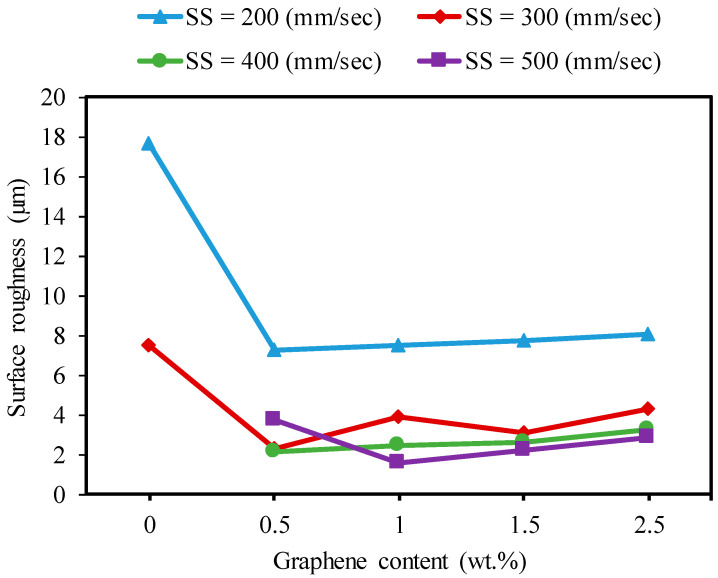
Effects of the graphene reinforcements and laser speed on the surface roughness during the micro-milling of the base Al_2_O_3_, and GNP/Al_2_O_3_ nanocomposites (F = 30 kHz).

Figure 17 illustrates the trend of the effect of graphene contents at different scanning speeds on the surface roughness of the fabricated microchannels. Regarding the impact of the graphene, it can be seen that the specimens with 0.3 wt.% and 1wt.% GNPs had the tendency to generate the lowest surface roughness. This trend was the same as that shown in Figure 14. That is, the surface roughness increased with the increasing channel depth for the nanocomposites with 0.5 wt.%, 1 wt.%, 1.5 wt.%, and 2.5 wt.% GNPs. This is because the increase in the surface roughness with the increase in the depth occurred partly due to more molten materials, and evaporation caused the redeposited material, which affected the generated finished surface [44]. Perrie et al. [50] found that the surface roughness of the ablated microchannel increases with the machined depth due to the material redeposition. In some cases, the surface roughness tended to decrease slightly with the GNPs increasing from 1 wt.% to 1.5 wt.%. It should be noted that the formation of the laser-machined surface was mainly governed by the molten and evaporated material and was negatively affected by the redeposition of the molten material on the ablated area. The base Al_2_O_3_ exhibited the worst surface roughness compared to all other samples. The molten material and plume of the Al_2_O_3_ rapidly moved forward and backward inside the channel, so the propagating laser beam was absorbed and blocked by them [51]. Hence, the surface roughness was increased due to increased redeposition of molten and evaporating material.

Regarding the effect of the scanning speed, Figure 17 shows that surface roughness significantly decreased with the scanning speed increasing from 100 mm/s to 500 mm/s for all samples in most of the machining conditions. This is because the laser energy per unit area decreased due to the reduced interaction time between the laser beam and material with an increase in the scanning speed. This resulted in fewer molten materials being deposited on the bottom surface [44].

**Figure 18:**
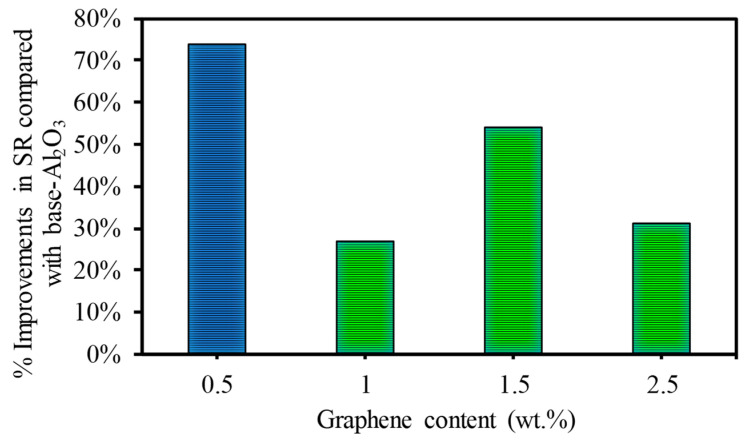
Percentage of improvement in surface roughness at a lower scanning speed of 200 mm/s.

It can be observed that the addition of GNPs enhanced the micromachining of alumina ceramic using a fiber laser with a minimum power of 20 watts. This was achieved by improving the surface quality at lower and higher scanning speeds. For example, at a lower scanning speed of 200 mm/s, the surface roughness levels decreased by 74%, 27%, 54%, and 31% for the 0.5 wt.%, 1 wt.%, 1.5 wt.%, and 2.5 wt.% GNP/Al_2_O_3_ nanocomposites, respectively, compared to the base Al_2_O_3_, as shown in Figure 18. At a higher scanning speed of 500 mm/s, higher-quality microchannels were fabricated on all GNP/Al_2_O_3_ nanocomposites. On the contrary, no ablated channels were fabricated on the base Al_2_O_3_ at the higher scanning speed of 500 mm/s.

**Figure 19:**
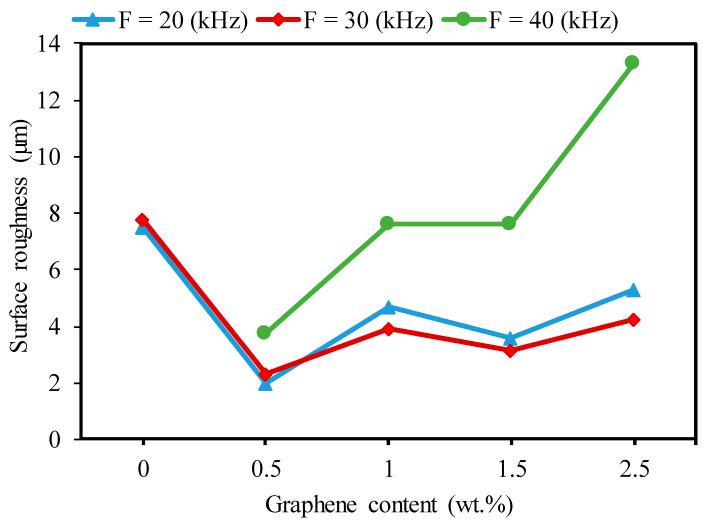
Effects of the graphene reinforcements and frequency on surface roughness during the micro-milling of the base Al_2_O_3_ and GNP/Al_2_O_3_ nanocomposites (SS = 300 mm/s).

Regarding the effects of the frequency, Figure 19 presents the effects of the GNP contents with varying frequencies on the surface roughness of the microchannels. It can be observed in Figure 19 that the surface roughness increased with increases in the GNP contents from 0.5 wt.% to 1 wt.% and then decreased when the GNP contents increased to 1.5 wt.%. After that, the surface roughness increased with increasing GNPs to 2.5 wt.%. This trend was almost the same as in the case of the channel depth shown in Figure 13a. This is attributed to differences in the material properties of the developed nanocomposites. The nanocomposites with 0.5 wt.% GNPs had the lowest surface roughness compared to the base Al_2_O_3_ and the 1 wt.%, 1.5 wt.%, and 2.5% GNP/Al_2_O_3_ by approximately 287%, 132%, 28%, and 165%, respectively, at a lower frequency of 20 kHz. The 2.5 wt.% GNP nanocomposite exhibited higher surface roughness than all the other specimens. This happened because of the lower ablation threshold and optical absorption in the case of the 2.5 wt.% GNP specimens, which led to a higher ablation depth with an increase in the surface roughness due to redeposited material on the bottom surface of the channel.

**Figure 20:**
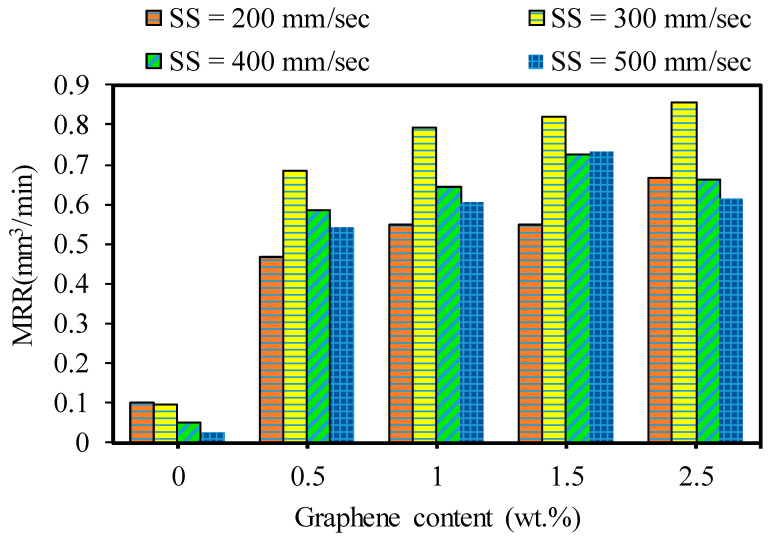
Effects of varying graphene contents with laser speed on MRR. At F = 30 kHz.

In general, it can be seen in Figure 19 that the surface roughness trend increased as the frequency increased from 20 kHz to 40 kHz for the 0.5 wt.% GNP/Al_2_O_3_. The low laser energy caused more unmolten materials to be deposited at the bottom of the fabricated microchannels, which affected the roughness of the ablated surface [40,49]. In addition, Figure 20 shows that the roughness decreased by increasing the frequency to 30 kHz from 20 kHz in the case of the 1 wt.%, 1.5 wt.%, and 2.5 wt.% GNP/Al_2_O_3_. This was expected due to their higher thermal resistance, smaller grain size, and lower light transmittance compared to the 0.5 wt.% GNP/Al_2_O_3_. At the frequency of 20 kHz, excessive energy was generated, and more materials were molten and evaporated, which led to a redeposit of molten material on the surface [49].

#### 3.2.4. Material Removal Rate

Figure 20 shows the effects of the GNP contents and laser scanning speed on the material removal rates of the base Al_2_O_3_ and GNP/Al_2_O_3_ nanocomposites. From the experimental findings, it was noted that the MRR was affected by the graphene contents. The MRR results are remarkably improved in the laser micromachining of all GNP/Al_2_O_3_ nanocomposites compared to the base Al_2_O_3_ ceramics. It can be observed in Figure 20 that the MRR values increased with increasing GNP contents from 0.5 wt.% to 1.5 wt.% in the micro-milling of the GNP/Al_2_O_3_ nanocomposites in most of the machining conditions. This is because the increase in the GNP content corresponded to lower light transmittance, higher thermal conductivity of the nanocomposite, and consequently, a higher ablation rate. In addition, this trend was almost the same as in the case of the microchannel depth. However, in the case of the 2.5 wt.% GNPs, the MRR decreased when the scanning speed increased to 500 mm/s from 300 mm/s compared to the 1.5 wt.% GNP specimens. This occurred because more material melted and was then deposited along the bottom and microchannel edges, which led to a change in the size of the machined channel (see Figure 9 and Figure 11).

In general, it can be seen in Figure 20 that the MRR tended to decrease as the scanning speed increased from 300 mm/s to 500 m/min for all fabricated samples. This is attributed to the fact that increasing the scanning speed led to reducing the interaction time between the laser and materials. Thus, the ablated materials decreased, resulting in a lower MRR, as discussed by [40,45]. In addition, it can be seen that the MRR increased when the scanning speed increased from 200 mm/s to 300 mm/s for all of the GNP/Al_2_O_3_ samples. Although the triangular microchannels were shaped at a lower scanning speed (200 mm/s), when the scanning speed was raised to 300 mm/s and higher, the shape of the microchannels changed from triangular to trapezoidal, with a large bottom width and less depth (see Figure 9 and Figure 11), which indicates a higher ablation rate compared to the lower scanning speed. These results are consistent with a previous study by [45].

**Figure 21:**
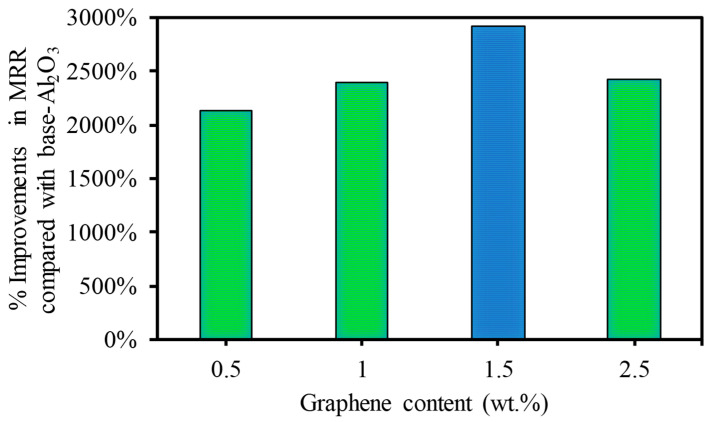
Percentages of improvement in MRR at a higher scanning speed of 500 mm/s.

To show the effects of adding GNP contents on the laser micromachining of Al_2_O_3_, Figure 21 is presented, which shows the improvement in the MRR of the GNP/Al_2_O_3_ nanocomposites compared to the base Al_2_O_3_. For example, at a lower scanning speed of 200 mm/s, the MRRs increased by 375.40%, 459.18%, 459.26%, and 581.788% for the 0.5 wt.%, 1 wt.%, 1.5 wt.%, and 2.5 wt.% GNP/Al_2_O_3_ nanocomposites, respectively, compared to the base Al_2_O_3_. At a higher scanning speed of 500 mm/s, the MRRs increased by 2134%, 2391%, 2915%, and 2427% for the 0.5 wt.%, 1 wt.%, 1.5 wt.%, and 2.5 wt.% GNP/Al_2_O_3_ nanocomposites, respectively, compared to the base Al_2_O_3_, as shown in Figure 21.

## 4. Conclusions

In this study, GNP/Al_2_O_3_ matrix nanocomposites consisting of 0 wt.%, 0.5 wt.%, 1 wt.%, 1.5 wt.%, and 2.5 wt.% GNPs were successfully fabricated using powder metallurgy and the HFIHS technique. The influence of the GNP contents on the micromachining performance of the fabricated nanocomposites was investigated. Based on the experimental findings, the following conclusions are drawn:

- All of the produced GNP-based Al_2_O_3_ nanocomposite samples exhibited high relative densities between 97.17% and 99.79%, which indicates good bonding between the GNPs and the Al_2_O_3_ matrix without porosity or cavities.
- The hardness was moderately affected by the GNP reinforcement in the Al_2_O_3_ matrix. Nanocomposites with 0.5 wt.% GNPs demonstrated a slight improvement in hardness by approximately 6.3% compared to the base Al_2_O_3_. In comparison, other nanocomposites exhibited a slight decrease in hardness.
- The SEM examination revealed that the inclusion of graphene contents had a profound influence on the surface morphology of the machined microchannels. The base Al_2_O_3_ samples showed inferior surface quality, with pores, more redeposited materials, and microcracks. All of the GNP/ Al_2_O_3_ nanocomposites showed improvement in morphology compared to the base Al_2_O_3_ samples. This was due to the lower ablation threshold energy of the graphene based-nanocomposites.
- The ablation depth was significantly affected by the GNP reinforcement. The GNP/Al_2_O_3_ nanocomposites exhibited improvement in the ablation depth compared to the base Al_2_O_3_ in all machining conditions. For example, at a scanning speed of 500 mm/s, the ablation depths increased by 9.8, 10.04, 10.9, and 14.6 times, respectively, compared to the base Al_2_O_3_. This was because the graphene reinforcements reduced the ablation threshold energy required to induce the materials and increased the material removal efficiency due to higher optical absorbance, thermal conductivity, and a smaller grain size.
- The MRRs during the laser micromachining were significantly affected by the GNP reinforcement in the Al_2_O_3_ matrix. For example, at a higher scanning speed, the MRRs were increased by 2134%, 2391%, 2915%, and 2427% for the 0.5 wt.%, 1 wt.%, 1.5 wt.%, and 2.5 wt.% GNP/Al_2_O_3_ nanocomposites, respectively, compared to the base Al_2_O_3_ ceramic.
- The roughness of the machined microchannels was affected by the GNP reinforcement. The nanocomposites with lower GNP contents exhibited the lowest surface roughness compared to the other samples. Among the machined nanocomposites, the 0.5 wt.% GNP samples showed the lowest surface roughness.
- Overall, the microchannel accuracy, surface quality, and material removal rate were significantly affected by the GNP reinforcement in the alumina matrix nanocomposites during the laser micromachining. It is worth stating again that all of the GNP-reinforced alumina matrix nanocomposites showed improved micromachining performance compared to the unreinforced samples. Moreover, by comparing the influence of the GNP reinforcements on the surface roughness and surface morphology, the nanocomposites with 0.3 wt.% and 1wt.% GNPs largely showed better performance in most of the machining conditions, while the nanocomposites with 1.5 wt.% and 2.5 wt.% GNPs showed better machining performance regarding the ablation rate and material removal rate. The results show that GNP/Al_2_O_3_ nanocomposites can be machined with very good quality using a very ordinary 20 W fiber laser. In contrast, pure Al_2_O_3_ could not be machined using the same low-power and low-budget laser system. This helps in achieving the clean and sustainable manufacturing goals with reduced energy consumption for clean environment.

## Figures and Tables

**Table 1:** The fabrication techniques of graphene-reinforced alumina ceramic nanocomposites presented in the literature.

	Ref.	CMC	Reinforcement Ratio	Preparation Method	Consolidation Method	Studied Characteristics	Machining Analysis
Graphene-based alumina matrix nanocomposites	[31]	GNS/Al_2_O_3_	3, 3.5, 4, 5, 10 and 15 vol.%	Dry ball milling	SPS	Electrical conductivity	No
	[25]	GNS/Al_2_O_3_	0.2, 0.5, 0.8,2 and 5 vol.%	Wet ball milling	SPS	Fracture toughness and elastic modulus	No
	[26]	GNS/Al_2_O_3_	0.1, 0.2, 0.5, and 1 wt.%	Wet ball milling	HP	Microstructure and fracture toughness	No
	[32]	GNP/Al_2_O_3_	0.5, 2, and 5 vol.%	Wet ball milling	SPS	Scratch testing	No
	[33]	GNP/Al_2_O_3_ CNT/Al_2_O_3_	1, 2 wt.%	Ultrasonic probe	HP and SPS	Morphology, grain sizes, and fracture mode	No
	[10]	GNS/Al_2_O_3_	0.25, 0.5, 1.5, 3 wt.%	Ultrasonic probe	HFIHS	Hardness, elastic modulus, and fracture toughness	No
	[34]	GNP/Al_2_O_3_	5, 10, 15, 20 vol.%	Wet ball milling	SPS	Hardness and electrical conductivity	Yes
	[29]	GNP/Al_2_O_3_	0.75, 1.17, 1.85, and 2.75 vol.%	Wet ball milling	Pressure-less sintering	Hardness, flexural strength, fracture toughness, and biocompatibility	No
	[35]	GNP/Al_2_O_3_	5, 10, 15 vol.%	Wet ball milling	SPS	Fracture toughness, wear resistance, and biocompatibility	No
	[30]	MLG/Al_2_O_3_	0.5, 1.0 vol.%	Aqueous sonic probe	HFIHS	Wear-resistance properties	No
	[13]	MLG/Al_2_O_3_	0.2, 0.5, 0.7, and 1 wt.%	Wet ball milling	SPS	Microstructure and tribological performance	No

**Table 2:** Composition of alumina powder.

Elements	Al_2_O_3_	B_2_O_3_	CaO	Fe_2_O_3_	MgO	Na_2_O
Percentage (wt.%)	≥99.9	≤0.002	≤0.01	≤0.01	≤0.02	≤0.03

**Table 3:** Characteristics of GNPs.

Powder	Average Diameter	Thickness	Surface Area	Density
GNPs	Less than 2 µm	5–8 nm	750 m^2^/g	2.21 g/cm^3^

**Table 4:** Laser parameters and their selected ranges.

Input Parameters	Values			
Scanning speed, SS (mm/s)	200	300	400	500
Pulse frequency, F (kHz)	20	30	40	-
Power, (w)	20	-	-	-
Scanning strategy	Line	-	-	-
Line spacing	17 µm	-	-	-
Spot diameter	50 µm	-	-	-
Pulse overlap	50%			
Step overlap	5%			

## Data Availability

The data are available on request from corresponding author.
